# A unique missense variant in the E1A-binding protein P400 gene is implicated in schizophrenia by whole-exome sequencing and mutant mouse models

**DOI:** 10.1038/s41398-021-01258-1

**Published:** 2021-02-18

**Authors:** Yoshiro Morimoto, Shinji Ono, Shintaro Yoshida, Hiroyuki Mishima, Akira Kinoshita, Takeshi Tanaka, Yoshihiro Komohara, Naohiro Kurotaki, Tatsuya Kishino, Yuji Okazaki, Hiroki Ozawa, Koh-ichiro Yoshiura, Akira Imamura

**Affiliations:** 1 Department of Neuropsychiatry, Unit of Translation Medicine, Nagasaki University Graduate School of Biomedical Sciences, Nagasaki, Japan; 2 Child and Adolescent Psychiatry Community Partnership Unit, Nagasaki University Hospital, Nagasaki, Japan; 3 Department of Human Genetics, Nagasaki University Graduate School of Biomedical Sciences, Nagasaki, Japan; 4 Department of Infectious Diseases, Nagasaki University Hospital, Nagasaki, Japan; 5 Department of Cell Pathology, Graduate School of Medical Sciences, Kumamoto University, Kumamoto, Japan; 6 Department of Clinical Psychology, Faculty of Medicine, Kagawa University, Kagawa, Japan; 7 Gene Research Center, Center for Frontier Life Sciences, Nagasaki University, Nagasaki, Japan; 8 Michino-o Hospital, Nagasaki, Japan

**Keywords:** Clinical genetics, Schizophrenia

## Abstract

Genetic and epidemiological evidence has suggested that genetic factors are important in schizophrenia, although its pathophysiology is poorly understood. This study used whole-exome sequencing to investigate potential novel schizophrenia-causing genes in a Japanese family containing several members affected by severe or treatment-resistant schizophrenia. A missense variant, chr12:132064747C>T (rs200626129, P2805L), in the E1A-binding protein P400 (EP400) gene completely segregated with schizophrenia in this family. Furthermore, numerous other EP400 mutations were identified in the targeted sequencing of a schizophrenia patient cohort. We also created two lines of Ep400 gene-edited mice, which had anxiety-like behaviours and reduced axon diameters. Our findings suggest that rs200626129 in EP400 is likely to cause schizophrenia in this Japanese family, and may lead to a better understanding and treatment of schizophrenia.

## Introduction

Schizophrenia is a severe and chronic neuropsychiatric disorder^1,2^ with numerous psychotic symptoms, including hallucinations and delusions^3–6^. Schizophrenia is usually treated with antipsychotics, although ~30% of cases respond poorly, which is termed treatment-resistant schizophrenia (TRS)^1,7^. Because of these clinical aspects, schizophrenia is a major cause of global disease burden^8^.

The pathophysiology of schizophrenia remains unclear. A number of genetic and epidemiological studies suggest that genetic factors play an important role in its pathogenesis, and its heritability estimate is 0.81^9^. Although genome-wide association studies (GWAS) have identified >150 schizophrenia-associated polymorphisms^10^, no specific variants with direct functional effects have been reported^11^. Furthermore, no common variant has an individually large effect, which is expected considering the selection pressures of impaired reproductive fitness associated with schizophrenia, and considering that the majority of risk cannot be accounted for by the associated genes reported to date^12,13^. Rare genetic variants detected by whole-exome sequencing (WES) or whole-genome sequencing (WGS) using next-generation sequencing (NGS) have demonstrated that the RNA-binding motif 12 (*RBM12*) gene, the SET domain containing 1 A (*SETD1A*) gene, and the solute carrier family 6 member 1 (*SLC6A1*) gene are high-risk factors^14–17^. The functional consequences of these rare variants are expected to be more readily interpretable^18^.

Using WES, the present study identified a unique missense variant in the E1A-binding protein P400 (*EP400*) gene in a Japanese family in which several members were affected by severe schizophrenia/TRS. Next, the frequencies of rare *EP400* variants were compared between sporadic schizophrenia cases and controls. We also generated *Ep400* gene-edited mice using clustered regularly interspaced short palindromic repeats (CRISPR)/Cas9. These mice developed anxiety-like behaviours and ultrastructural abnormalities in the central nervous system (CNS). We report here an *EP400*/*Ep400* variant that elicited abnormalities in neurobehaviour, which may lead to schizophrenia.

## Subjects and methods

### Clinical presentation in a Japanese family

**Fig. 1:**
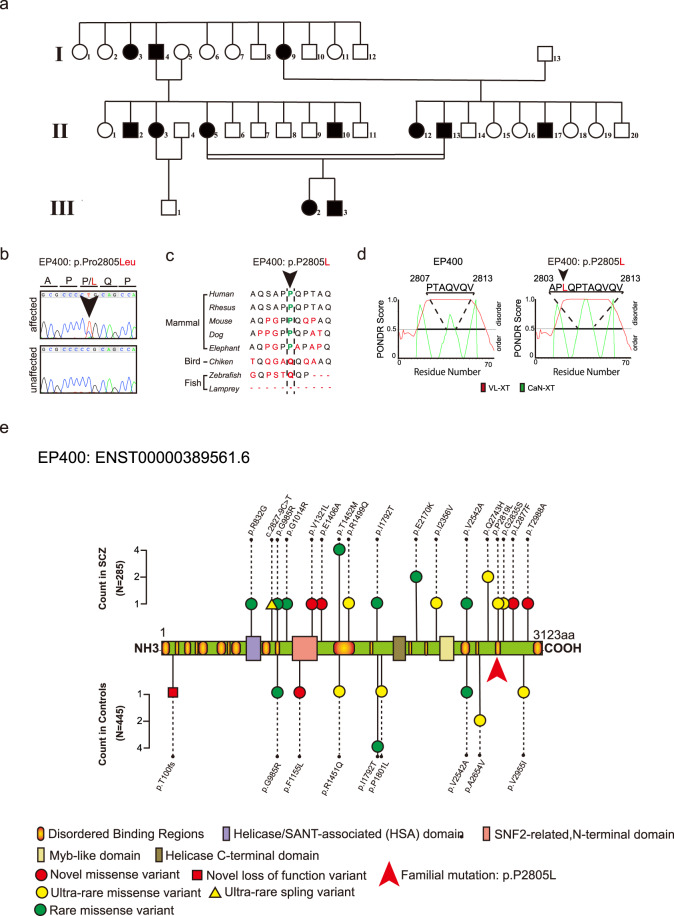
Japanese family with multiple individuals affected by schizophrenia. **a** Japanese family with multiple individuals affected by schizophrenia. Black symbols represent individuals diagnosed with schizophrenia. **b** Segregation of the missense mutation (chr12: 132064747; C>T on hg38, p.P2805L in ENST00000389561.7) in *EP400* with schizophrenia. **c** The position of the familial missense mutation in the *EP400* gene. **d** The CaN-XT algorithm in PONDR was used to predict the effect of p.P2805L on the EP400 protein. **e** *EP400* deleterious variants in Japanese individuals with or without schizophrenia. Rare variants are represented by lollipops, and counts of alleles with variants in the cases (top panel) and controls (bottom panel) are shown. Novel variant = novel variant in all databases; ultra-rare variant = variants with MAF < 0.001 in any database; rare variant = variants with 0.001 < MAF < 0.005 in any database.

Three first-generation family members were diagnosed with schizophrenia. One (Fig. 1a, I-9) had consulted a psychiatric hospital because of auditory hallucinations and delusions at 47 years old and was diagnosed with schizophrenia. Her elder sister and brother (I-3 and I-4, respectively) also had auditory hallucinations and delusions and were hospitalised more than once; however, their medical records were not preserved. Seven second-generation family members (II-2, II-3, II-5, II-10, II-12, II-13, and II-17) were diagnosed with schizophrenia and treated with antipsychotic medications. Two of these second-generation patients (II-3 and II-17) were diagnosed with TRS^19^ and required multiple long-term hospitalisations, but were not treated with clozapine or electroconvulsive therapy. Furthermore, two third-generation family members were diagnosed with schizophrenia (III-2 and III-3). III-2 refused medical care and medication. None of the patients had neurological abnormalities by standard neurological exams or comorbidities. All subjects in the family were evaluated by two or more psychiatrists. Diagnoses of psychiatric diseases were made with reference to the International Classification of Diseases, Tenth Revision (ICD-10) and the Diagnostic and Statistical Manual of Mental Disorders, Fifth Edition (DSM-5). A detailed clinical presentation of the Japanese family is summarised in Table 1.

**Table 1:** Clinical presentation of the Japanese family including multiple patients.

ID	Age of onset (years)	Diagnosis		Clinician-rated dimensions of psychosis symptom severity					GAF	Medications	Other
		ICD-10	DSM-5	I	II	III	IV	V			
I-9	47	F20.00	295.90	2	2	3	1	3	45	HPD^a^	–
II-3	21	F20.00	295.90	3	3	4	1	4	15	HPD, ZTN, CHP, OLZ, QUE, RIS	TRS
II-5	34	F20.04	295.90	3	2	2	1	3	45	RIS*, OLZ, QUE	–
II-12	18	F20.00	295.90	2	2	2	0	3	45	HPD*, OLZ, QUE, RIS	–
II-17	17	F20.10	295.90	3	3	4	2	4	15	HPD, OLZ, QUE, RIS	TRS
III-2	17	F20.10	295.90	1	1	3	1	4	35	–	No consent for drug treatment
III-3	30	F20.01	295.90	2	2	1	0	2	65	OLZ^a^	–

*Diagnosis*: ICD-10: International Statistical Classification of Diseases and Related Health Problems, 10^th^ edition, DSM-5: Diagnostic and Statistical Manual of Mental Disorders, 5th Edition; F20.00: paranoid schizophrenia, continuous; F20.01: paranoid schizophrenia, episodic with progressive defect; F20.04: paranoid schizophrenia, incomplete remission; F20.10: hebephrenic schizophrenia, continuous; 295.90: schizophrenia. *Clinician-Rated Dimensions of Psychosis Symptom Severity***:** I: hallucinations, II: delusions, III: disorganised speech, IV: abnormal psychomotor behaviour, V: negative symptoms. **GAF**: Global Assessment of Functioning Scale. *Medications*: HPD: haloperidol, ZTN: zotepine, CHP: chlorpromazine, OLZ: olanzapine, QUE: quetiapine, RIS: risperidone. *Other***:** TRS: treatment-resistant schizophrenia.

^a^Drugs that improved the GAF scale over 40.

### DNA extraction

Of the 10 family members recruited, 7 were affected (I-9, II-3, II-5, II-12, II-17, II-2, and III-3) and 3 were unaffected (I-13, II-11, and II-20) by schizophrenia (Fig. 1a). After obtaining written consent, 10 mL of peripheral blood was collected from individuals. Genomic DNA was extracted from peripheral blood leukocytes using a QIAamp DNA Midi Kit (Qiagen, Hilden, Germany).

### WES

We assessed DNA quality using a NanoDrop 2000 (Thermo Fisher Scientific, Waltham, MA, USA) and a Qubit fluorometer for nucleic acid quantification (Thermo Fisher Scientific). We performed WES for the seven affected and three unaffected individuals (Fig. 1a) using a SureSelect Exome Target Enrichment System v5 (Agilent Technologies, Santa Clara, CA, USA) followed by paired-end sequencing on a HiSeq 2500 (Illumina, San Diego, CA, USA). Mean depths and coverage rates are summarised in Supplementary Table 1.

### Sequencing data analysis

Fastq format files were generated using bcl2fastq software. Novoalign software (Novocraft Technologies, Kuala Lumpur, Malaysia) was used to align reads on the hg38/GRCh38 human reference genome sequence. Aligned reads were sorted using Novosort software (Novocraft Technologies) and subjected to polymerase chain reaction (PCR) marking and optical duplication using MarkDuplicates in the Picard tools package (http://broadinstitute.github.io/picard/). The Genome Analysis Toolkit (GATK)^20^ was used to perform local realignment (GATK IndelRealigner), and variant calling (GATK HaplotypeCaller) was implemented in an in-house workflow management tool^21^. Single nucleotide variations (SNVs) and insertions/deletions (indels) were annotated using ANNOVAR software^22^.

### WES variant filtering

Substitutions meeting the following criteria were considered ‘deleterious’: (1) mutations: stop-gain, stop-loss, nonsynonymous, or splice-site mutations according to GENCODE basic 33, downloaded from the UCSC Genome Browser (https://genome.ucsc.edu); and (2) alternative allele frequencies of ≤0.5% at mutation loci in the following databases: 4.7KJPN data of allele and genotype frequency panels from 4773 Japanese individuals (https://jmorp.megabank.tohoku.ac.jp/202001/variants); genome aggregation database (gnomAD) v2.1.1 and v3 (https://gnomad.broadinstitute.org);^23^ and Human Genomic Variation Database (HGVD) v2.3 exome data from Japanese individuals (http://www.hgvd.genome.med.kyoto-u.ac.jp/index.html)^24^. Two deleterious variants were shared among the affected individuals (Supplementary Table 2). We excluded rs768213554 in strawberry notch homolog 1 (*SBNO1*) gene from the candidates because it was found in the cohort of 445 Japanese controls. Consequently, we considered the rs200626129 variant in *EP400* as the potential pathogenic variant of our family. The WES variant filtering process is summarised in Supplementary Table 3.

### Sanger sequencing

Sanger sequencing confirmed that one variant was shared among all of the affected individuals and was absent in the three unaffected individuals. Primers were designed using Primer3:^25^ chr12:132064747; C>T, forward, 5′-TCATCAAAATGCAGAAGCAGA-3′, reverse, 5′-TCATCAAAATGCAGAAGCAGA-3′. Genomic DNA (5 ng) was amplified using KOD FX Neo polymerase (Toyobo, Osaka, Japan) by PCR in 20-µL reactions. PCR products were purified using AMPureXP® (Agencourt, Beverly, MA, USA) and sequencing reactions were performed using a BigDye Terminator Cycle Sequencing Kit v3.1 (Applied Biosystems, Foster City, CA, USA). After purification using CleanSEQ (Agencourt), DNA fragments were run and separated on an ABI Genetic Analyzer 3130 (Applied Biosystems).

### Mutational analysis of Japanese inpatient schizophrenia case–control groups

Samples from 285 sporadic, inpatient schizophrenia patients in Japan, who had been diagnosed by psychiatrists in accordance with the DSM-5 or ICD-10, and 445 control samples were collected. For the schizophrenia association test using variants in the *EP400* locus, we performed target sequencing using a SeqCap EZ Choice Library (Roche NimbleGen, Madison, WI, USA) according to the manufacturer’s protocol. Prepared libraries were sequenced using a HiSeq2500 (Illumina). Subsequent analyses were performed using the hg19/GRCh37 human reference genome sequence, following the “sequencing data analysis” section. CrossMap (http://crossmap.sourceforge.net)^26^ was used to convert data to hg38/GRCh38 human reference genome sequence assemblies.

### In silico analysis of rs200626129 in *EP400*

To analyse potential functional changes in the EP400 protein, mutation significances were scored using SIFT^27^, PROVEN^28^, MutationTaster^29^, and M-CAP^30^. The Predictor of Natural Disordered Regions (PONDR) algorithm was used to predict disordered protein regions (http://www.pondr.com/pondr-tut1.html)^31–33^.

### Single-locus association test with deleterious variants

To identify unique deleterious variants in *EP400* that were associated with schizophrenia, we tested the accumulation of 23 deleterious variants identified by target sequencing. We used Fisher’s exact test to estimate *P*-values. The significance threshold was 0.002174, with Bonferroni correction.

### Gene-based rare-variant association tests

To test differences in the burden of the candidate gene, we used Efficient and Parallelizable Association Container Toolbox (EPACTS) algorithms, which provide three different analyses (combined multivariate and collapsing [CMC]^34^, Madsen–Browning^35^, and optimal sequence kernel association test [SKAT-O]^36^). We excluded common variants with alternative allele frequencies > 0.005 in any public database (1000 Genomes Project, all population data released October 2014;^37^ NIH NHLBI 6 515 exome data [http://evs.gs.washington.edu/EVS/]; Exome Aggregation Consortium 65 000 exome data;^38^ HGVD exome data of 1 208 individuals in Japan;^39^ and SNV allele frequency in whole-genome sequencing data of 2049 healthy Japanese individuals [https://ijgvd.megabank.tohoku.ac.jp]) and >0.005 in our in-house control samples, with a call rate <90% or genotyping quality <99. We selected four histone-modifying genes (the histone deacetylase 4 [*HDAC4*] gene, the lysine methyltransferase 2D [*KMT2D*] gene, the lysine demethyltransferase 6A [*KDM6A*] gene, and the nuclear receptor binding SET domain protein 1 [*NSD1*] gene) as controls. The significance threshold was set to 0.01, with Bonferroni correction.

### Generation of *Ep400* knock-in mice

We designed guide RNA onto exon 47 of mouse ortholog *Ep400*, corresponding to the mutation position in the family from the present study. Single-guide RNA was designed using the ATUM gRNA Design Tool (https://www.atum.bio/eCommerce/cas9/input) as follows: 5′-CACTTGCGCTGGTTGCTGTG-3′. Single-strand DNA oligonucleotides (ssODN) to introduce mutations into mouse DNA were as follows: #1: 5′-CCACCACAGCCCCCACCACCGCAGGCGCAGCCAGGTCCCCTACAGCAACCAGCGCAAGTGCAAGTACAGACTCCACAGCC-3′, #2: 5′-GATGCAGCTGCCACCACAGCCCCCACCACCGCAGGCCCAGCCAGGTCCTCTACAGCAACCAGCGCAAGTGCAAGTACAGACTCCACAGCCCCCACAGCAA-3′.

Single-guide RNA, crRNA, tracrRNA, HiFi Cas9 (IDT Inc, Coralville, IA, USA), and ssODN (FASMAC, Kanagawa, Japan) were mixed according to the mouse zygote microinjection protocol provided by IDT Inc., and were then injected into C57BL/6N zygotes using a microinjector (Olympus IX70 Fluorescence Microscope Cutaway, Olympus, Tokyo, Japan), TransferMan NK2 (Eppendorf, Hamburg, Germany), FemtoJet 4i (Eppendorf), and CellTram Oil (Eppendorf). Next, zygotes were cultured until the two-cell stage in vitro and transferred to recipient pseudopregnant female oviducts. PCR was used to verify genotypes from mouse DNA using KOD FX Neo polymerase (Toyobo). The PCR primers were as follows: forward, 5′-TGGAGGGTGGCTTATGGTTA-3′; reverse, 5′-GTCACTGTGGTGCCTGTGAG-3′.

### Histological analysis

Fresh mouse brains and spinal cords were fixed in 10% formalin for 72 h, embedded in paraffin, and sectioned at 5-µm intervals. After deparaffinisation, slides were stained with haematoxylin and eosin (HE) to visualise structures. The Klüver–Barrera (KB) stain was used to detect myelin sheaths. We viewed and photographed sections using a BZ-9000 All-in-One Fluorescence Microscope (Keyence, Osaka, Japan). For immunohistochemical staining, tissue slides were deparaffinised, rehydrated, and rinsed in Tris-buffered saline (TBS). For antigen retrieval, tissue sections were microwaved for 20 min in 10 mM citrate buffer using an MI-77 microwave processor (Azumaya Corporation, Tokyo, Japan). Sections were then blocked in Protein Block Serum-Free (Dako, Carpinteria, CA, USA) for 1 h at room temperature, followed by incubation with primary and secondary antibodies for 1 h each at room temperature. Immunofluorescent staining was performed using the following primary antibodies: polyclonal anti-EP400 (HPA016704, Atlas Antibodies, Bromma, Sweden) and polyclonal anti-MAP2 (ab5392, Abcam, Cambridge, UK). Secondary antibodies were goat anti-chicken IgY H&L Alexa Fluor 488 (ab150169, Abcam) and goat anti-rabbit IgG H&L Alexa Fluor 555 (ab150078, Abcam). Primary and secondary antibodies were diluted using Antibody Diluent with Background Reducing Components (Dako). Sections were mounted with Vectashield Mounting Medium for fluorescence analysis with DAPI (Vector Laboratories, Burlingame, CA, USA). Fluorescent images were taken using a BZ-9000 All-in-One Fluorescence Microscope (Keyence).

### Transmission electron microscopy (TEM)

Samples were fixed with 2% glutaraldehyde (Nacalai Tesque, Kyoto, Japan) in 0.1 M sodium cacodylate buffer containing 1 mM CaCl_2_ and 1 mM MgCl_2_ (cacodylate buffer, pH 7.4) at room temperature for 72 h, rinsed with cacodylate buffer, and post-fixed with 1% OsO_4_ (Nacalai Tesque) in cacodylate buffer at 4 °C for 60 min. Next, samples were washed with cacodylate buffer, dehydrated in a graded series of ethanol and acetone, and embedded in Quetol 651 epoxy resin (Nisshin EM, Tokyo, Japan). The blocks were then sectioned at 5-µm intervals and stained with toluidine blue (TB) to visualise myelin sheaths. Resin-embedded samples were further trimmed and sectioned using a diamond knife on an ultramicrotome (Reichert-Jung, Vienna, Austria). Ultra-thin sections were collected on grids, stained with uranyl acetate and lead citrate, and examined at 80 kV by TEM (JEM-1230; JEOL, Tokyo, Japan). Axon diameters and G-ratios of randomly selected axons in the white matter of the spinal cord were measured using ImageJ (axon diameter, *n* = 100; G-ratio, *n* = 30)^40,41^. The two-sided *F*-test was applied to confirm normally distributed data. The two-sided Student’s *t*-test and two-sided Welch’s *t*-test were applied for comparison. The significance threshold was set to 0.05.

### Neurobehavioural screening battery in *Ep400* knock-in mice

The mice used in the present study were derived from backcrossing to C57BL/6N for five generations. Six male Ep400-p.P2715L#1 mice and six male wild-type (WT) littermates were examined using behavioural tests at 3 and 6 months old. Mice were housed individually at 22 °C–26 °C, humidity 40%–65%, and 12-h light/dark periods. *Hindlimb clasping*: a marker of disease progression in a number of mouse models of neurodegeneration^42,43^. We evaluated hindlimb clasping scores using the following criteria^44^. Hindlimbs were consistently splayed outward, away from the abdomen = 0. One hindlimb was retracted toward the abdomen for more than 50% of the time suspended = 1. Both hindlimbs were partially retracted toward the abdomen for more than 50% of the time suspended = 2. Hindlimbs were entirely retracted and touching the abdomen for more than 50% of the time suspended = 3. *Open-field test***:** measures locomotor activity, exploratory drive, neophobia, and certain aspects of anxiety^45^. Mice were placed in a clean box measuring 50 × 50 × 40 cm. The central area was 60% of the area. After 15 min resting time, mice were placed peripherally in the box facing outward, and were observed for 10 min by video tracking. Video data were analysed using Bara-Baby X (LNSOFT, Tokyo, Japan). Migration length (cm) was measured as horizontal activity. Centre time was calculated as the ratio of movement time in the central area to the entire movement time. *Social interaction test***:** evaluates social behaviour phenotypes by placing two animals in a neutral cage and measuring the percentage of time spent in direct physical contact, including ano-genital exploration, sniffing with direct contact, crawling, grooming, and play behaviour^46^. Mice were kept in cages for 7 days to prepare, and stimulator mice were then put into the same cage as the subject mice under video monitoring. After 15 min, stimulator mice were removed, and video monitoring was stopped. Movies were analysed using BORIS Software 4.1.4 (https://www.boris.unito.it)^47^. *Y-maze test***:** evaluates short-term spatial working memory^48^. After 15 min resting time, mice were placed on one corner of an arm of the apparatus, facing the outer side, under video monitoring. Mice were monitored for 10 min without stimulation, and the number of entries into each of three arms was counted. Spontaneous alternations were calculated if the mouse entered three different arms sequentially. *Rotarod test*: evaluates motor coordination^49^. Mice in a “stable state” after 20 min rest were placed on the rotarod (Rota-Rod Treadmill for Mice, Model MK-610A, Muromachi Kikai, Tokyo, Japan) at 4 rpm. After several seconds, the rotor accelerated to 40 rpm for 300 s. Tests were repeated up to three times if mice fell off before 10 s had passed (Trial 1). After 20 min, the same tests were again administered (Trial 2). An two-sided *F*-test confirmed normally distributed data and two-sided　Student’s *t*-test was used for two-sample significance tests. The significance threshold was set to 0.05. All behavioural testing was performed by Unitech Co., Ltd. (Chiba, Japan).

## Results

### WES and variant filtering

We identified a large Japanese family with autosomal dominant inheritance of schizophrenia. The patients in this family exhibited severe negative symptoms and low scores in the Global Assessment of Functioning Scale (Clinician-Rated Dimensions of Psychosis Symptom Severity: V_average_ = 3.29; Global Assessment of Functioning Scale: GAF_average_ = 37.9). Of the patients in this family, two second-generation TRS patients (II-3 and II-17) responded poorly to antipsychotics and required multiple long-term hospitalisations (Table 1). To identify potential pathogenic genetic variants, we performed WES on 10 family members (affected: I-9, II-3, II-5, II-12, II-17, III-2, III-3; unaffected: I-13, II-11, II-20; Fig. 1a). Using NGS, we detected 148,069 variants, of which 2176 variants were annotated to be deleterious (Supplementary Table 3). Of the 2 176 deleterious variants, only two variants, on chromosome 12q24.3, were completely segregated with schizophrenia (chr12:123311084, T>C [rs768213554] in *SBNO1*; and chr12:132064747, C>T [rs200626129] in *EP400*; Supplementary Tables 2, 3). Next, we screened these two substitutions in 445 healthy Japanese individuals and found that rs768213554 in *SBNO1* gene was present in healthy controls, as well as in two Japanese public databases (control cohort: minor allele frequency [MAF] = 0.0022; HGVD v2.30: MAF = 0.00124; 4.7KJPN: MAF = 0.0012). Because the pathogenic mutation in this family was expected to have an extremely high effect size and penetrance rate of Mendelian inheritance, we excluded rs768213554 in *SBNO1* from the candidate variants. Notably, mutation screening indicated that rs200626129 in *EP400* (confirmed by direct sequencing; Fig. 1b) was a ‘unique’ variant, because it was a novel variant in both healthy controls and Japanese databases (control cohort: MAF = none; HGVD2.30: MAF = none; 4.7KJPN: MAF = none; Supplementary Tables 2, 3).

The position of the familial mutation, in exon 48 of the *EP400* gene, was located within a disordered binding region that is highly conserved among mammals (Fig. 1c). We next analysed whether rs200626129 in *EP400* affects protein function, using SIFT, PROVEN, MutationTaster, and M-CAP. All programs evaluated the variant as ‘damaging’ (SIFT score = 0.007, deleterious; PROVEN score = −2.67, deleterious; MutationTaster score = 0.994, disease-causing; M-CAP score = 0.254, damaging). The CaN-XT algorithm in PONDR predicted that the variant would elongate the disordered region of the EP400 protein (Fig. 1d). These findings suggest that rs200626129 in *EP400* causes dysfunction of the protein, and that this variant is relevant to the pathogenesis of schizophrenia.

The rs200626129 variant in *EP400* was considered to be the pathogenic variant in this family because of its uniqueness and complete linkage, although its genetic burden in the development of schizophrenia and its pathogenic effects on CNS functions were not evaluated.

### Single-locus association test with the deleterious variants identified by *EP400* sequencing

To evaluate the MAF of rs200626129 in sporadic schizophrenia, and to identify other unique deleterious variants in *EP400* that are associated with schizophrenia, we performed target capture sequencing of *EP400* in Japanese individuals with or without schizophrenia (schizophrenia: *n* = 285; controls: *n* = 445). Notably, rs200626129 in *EP400* was not identified in the 285 schizophrenia patients.

Nevertheless, we identified 23 deleterious variants in *EP400* using targeted sequencing. Of these 23 variants, 6 were novel variants that were not registered in databases (HGVD2.30, 4.7KJPN, or gnomAD exome), 10 were ultra-rare variants that were registered in at least one database with MAF < 0.001, and 7 were rare variants registered in at least one database with 0.001 < MAF < 0.005. None of the 23 single loci had a significant association with schizophrenia (Fig. 1e, Supplementary Table 4).

### Gene-based rare-variant association tests

Schizophrenia patients had a tendency toward a higher frequency of deleterious variants (7.37%) compared with controls (3.15%; Table 2). To confirm the higher frequency of deleterious variants in *EP400* in patients with schizophrenia compared with controls, we performed gene-based rare-variant association tests using EPACTS algorithms. We selected four histone-modifying genes (*HDAC4*, *KMT2D*, *KDM6A*, and *NSD1*) as controls. In the gene-based rare-variant association tests, *EP400* showed the lowest *P*-values in the five tested genes, but did not show significant enrichment of rare variants in schizophrenia (CMC, *P* = 0.059184; Madsen–Browning, *P* = 0.051225; SKAT-O, *P* = 0.010889; Table 2).

**Table 2:** Rare variants in gene-based burden tests for *EP400*.

Gene	Count of rare deleterious variants		Frequency of rare variants in SCZ subjects (*n* = 285)	Frequency of rare variants in control subjects (*n* = 445)	*P*-value		
	SCZ (*n* = 285)	Controls (*n* = 445)			CMC	Madsen–Browning	SKAT-O
*EP400*	21	14	7.37%	3.15%	0.059	0.051	0.011
*HDAC4*	6	8	2.11%	1.80%	0.955	0.959	0.36
*KMT2D*	35	37	12.28%	8.31%	0.358	0.288	0.194
*KDM6A*	9	7	3.16%	1.57%	0.304	0.295	0.395
*NSD1*	13	18	4.56%	4.04%	0.644	0.648	0.834

The significance threshold was 0.01, with Bonferroni correction. SCZ: schizophrenia; CMC: combined multivariate and collapsing; SKAT-O: optimal sequence kernel association test.

### Generation of Ep400-p.P2715L mice using CRISPR/Cas9 technology

**Fig. 2:**
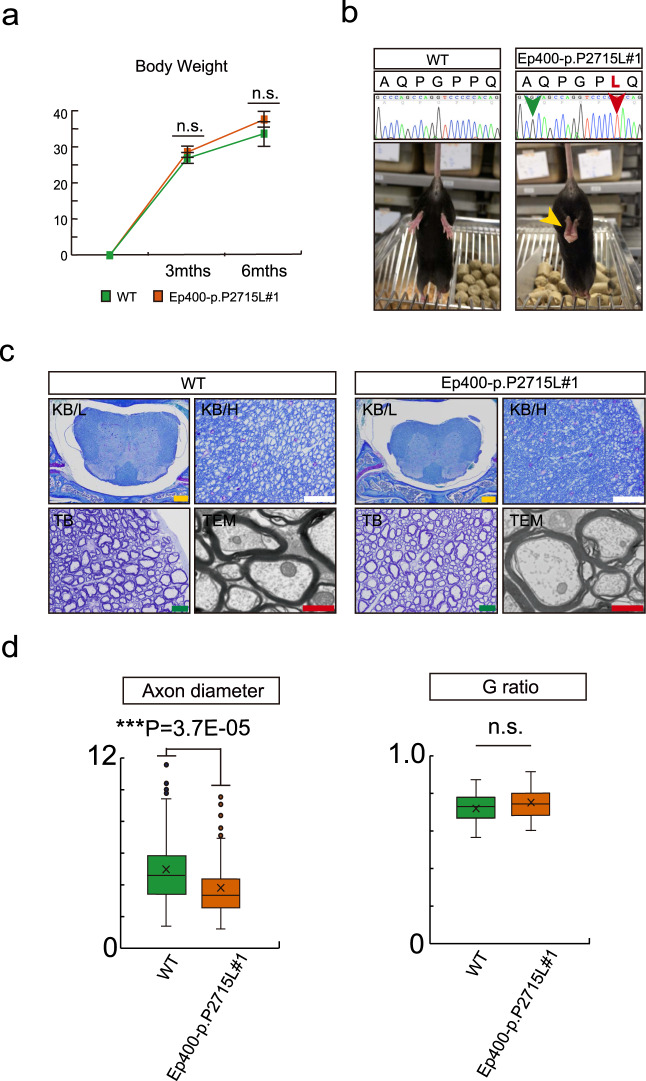
Screening analysis of known phenotypes in Ep400-p.P2715L mice. **a** Body weight of Ep400-p.P2715L#1 mice and their WT littermates (p.P2715L#1 mice: *n* = 6, WT: *n* = 6). There was no significant difference in body weight in adult mice. **b** Genotype and hindlimb clasping. Sanger sequencing was used to confirm the missense mutation (p.P2715L, red arrow) and synonymous mutation to break the protospacer adjacent motif (*PAM*; green arrow) in *Ep400*. Hindlimb clasping (yellow arrow) was used as a marker of CNS dysfunction (p.P2715L#1 mice: *n* = 6, WT: *n* = 6). **c** In a low-power field, there was no obvious differences in KB-stained tissue between 3-month-old Ep400-p.P2715L#1 mice and their WT littermates. Ep400-p.P2715L#1 mice showed a decrease in axon diameters in the spinal marrow by both KB and TB staining in a high-power field. Ep400-p.P2715L#1 mice did not show any morphological abnormalities in myelin sheaths in the TEM analysis (p.P2715L#1 mice: *n* = 2, WT: *n* = 2). KB/L = Klüver–Barrera staining, in a low-power field. KB/H = Klüver–Barrera staining, in a high-power field. TB = Toluidine blue staining. Yellow scale bar = 200 µm. White scale bar = 50 µm. Green scale bar = 10 µm. Red scale bar = 1 µm. **d** TEM analysis was used to compare axon diameters and G-ratios between Ep400-p.P2715L#1 and WT mice. A significant decrease in axon diameter in Ep400-p.P2715L#1 mice was confirmed by TEM analysis. Ep400-p.P2715L#1 mice did not show significant differences in G-ratios compared with their WT littermates (p.P2715L#1 mice: *n* = 2, WT: *n* = 2).

To reveal the functional significance of the familial mutation in brain morphology and behavioural characteristics, we generated two lines of Ep400^P2715L/P2715L^ mice (Ep400-p.P2715L#1 and #2), corresponding to the human P2805L mutation, using CRISPR/Cas9. The Ep400-p.P2715L#1 mice were born at a predicted ratio by Mendelian segregation (Supplementary Table 5) and did not show any differences in body size compared with controls (3 months old: Student’s *t*-test, *P* = 0.1304; 6 months old: Student’s *t*-test, *P* = 0.07203; Fig. 2a, Supplementary Table 6). According to public databases, the EP400 protein is expressed in the brains of both mice and humans^50,51^. Furthermore, our immunofluorescent staining results confirmed the expression of EP400 in 3-month-old mouse brains, especially in neural cell nuclei. However, there were no differences in EP400 protein expression patterns between the knock-in mice and their WT littermates (Supplementary Fig. 1).

### Screening analysis for the known phenotypes of *Ep400* conditional-knockout mice (hindlimb clasping and myelin abnormalities) using Ep400-p.P2715L mice

Recent studies have reported distinctive phenotypes in *Ep400* conditional-knockout mice (notably, hindlimb clasping and myelin abnormalities)^52,53^. Thus, to investigate the possibility that the p.P2715L mutation also causes Ep400 protein dysfunction in vivo, we screened our Ep400-p.P2715L mice for these known phenotypes of *Ep400* conditional-knockout mice.

The Ep400-p.P2715L#1 mice showed peculiar hindlimb clasping from 3 months of age (hindlimb clasping score, WT_average_ = 0.5; Ep400-p.P2715L#1_average_ = 1.833; Student’s *t*-test, *P* = 0.046; Fig. 2b, Supplementary Table 7, Supplementary Video 1), which is a sign that is observed in mice with CNS dysfunction and is used as a marker of disease progression in a number of mouse models of neurodegeneration^42,43^. This behaviour was also present in Ep400-p.P2715L#2 mice (hindlimb clasping score, Ep400-p.P2715L#2_average_ = 2; Student’s *t*-test, *P* = 0.023; Supplementary Fig. 2a, Supplementary Table 7).

EP400 is expressed in all cell types in the developing rodent CNS, and EP400-dependent SWR chromatin remodelling activity is required for myelination. Hypomyelination and myelin abnormalities have been previously reported in the central and peripheral nervous systems of oligodendrocyte-specific *Ep400* conditional-knockout mice^52,53^. Of note, we observed decreased axon diameters in the knock-in mice compared with the WT mice in KB and TB staining. However, there were no morphological abnormalities in the myelin sheaths of Ep400-p.P2715L#1 mice (Fig. 2c). Axon diameters were significantly decreased in the Ep400-p.P2715L#1 mice under TEM (WT_average_ = 4.9313 µm, Ep400-p.P2715L#1_average_ = 3.735 µm; Welch’s *t*-test, *P* = 3.71769E-05; Fig. 2d). However, TEM analysis indicated no differences in G-ratios (diameter of naked axon/diameter of myelinated axon) in the Ep400-p.P2715L#1 mice compared with their WT littermates (WT_average_ = 0.7143, Ep400-p.P2715L#1_average_ = 0.7506; Student’s *t*-test, *P* = 0.0732; Fig. 2d). The histological features of Ep400-p.P2715L#2 mice were the same as those of Ep400-p.P2715L#1 mice (Supplementary Fig. 2b, c).

### HE staining of CNS tissue from Ep400-p.P2715L mice

We used histological methods to examine morphological abnormalities in the CNS of Ep400-p.P2715L#1 mice, and observed no clear differences (e.g., abnormalities in cortical structure, neuronal dropout, or cortical atrophy) in HE-stained tissue from these mice compared with their WT littermates (Supplementary Fig. 3a, b).

### Neurobehavioural screening battery in Ep400-p.P2715L mice

**Fig. 3:**
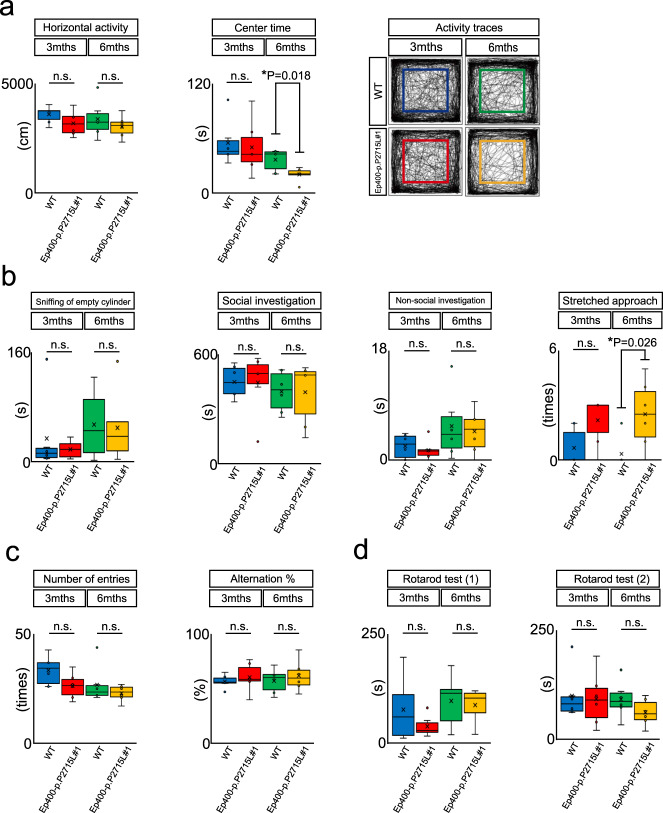
Neurobehavioural screening battery in *Ep400* knock-in mice. Ep400-p.P2715L#1 mice and their WT littermates were examined using behavioural tests (p.P2715L#1 mice: *n* = 6, WT: *n* = 6). **a** Open-field test. Horizontal activity and centre time were measured, and activity traces are shown. The coloured squares represent the centre areas. **b** Social interaction test. Various social interaction behaviours were measured and compared. **c** Y-maze test. The number of entries and the percentage of alternations were measured and compared. **d** Rotarod test. Time spent on the rotarod before falling was measured and compared.

To date, there have been no reports of the effects of *Ep400* dysfunction on neurobehavioural phenotypes. Thus, to evaluate the potential impact of EP400-p.P2715L protein expression in the CNS on neurobehavioral phenotypes, we screened for neurobehavioural abnormalities using a neurobehavioural screening battery, which consisted of four different tests (the open-field, Y-maze, rotarod, and social interaction tests). The open-field test showed no significant differences in behaviour between 3-month-old Ep400-p.P2715L#1 mice and WT littermates. However, 6-month-old Ep400-p.P2715L#1 mice showed significantly decreased central time (WT_average_ = 36.8 s, Ep400-p.P2715L#1_average_ = 20.4 s; Student’s *t*-test, *P* = 0.018; Fig. 3a). The social interaction test also revealed no significant differences between 3-month-old Ep400-p.P2715L#1 mice and WT littermates. In contrast, 6-month-old Ep400-p.P2715L#1 mice hesitated to approach the stimulator mice and showed a significantly increased number of stretched approaches compared with WT littermates (WT_average_ = 0.333, Ep400-p.P2715L#1_average_ = 2.5; Student’s *t*-test, *P* = 0.0265; Fig. 3b, Supplementary Video 2). These data suggest that Ep400-p.P2715L#1 mice develop anxiety-like behaviours with age. The Ep400-p.P2715L#1 mice showed no significant behavioural abnormalities in the Y-maze or rotarod tests at any age (Fig. 3c, d).

## Discussion

We identified a possible novel schizophrenia-causing gene in a Japanese family containing multiple members affected with severe schizophrenia/TRS. Using WES, a unique missense variant in *EP400* was identified that completely segregated with disease in this family.

Few studies have reported an association between *EP400* and psychiatric disorders. Prados et al. reported that a DNA methylation status change of *EP400* was associated with borderline personality disorder^54^. However, no study has yet reported an association between *EP400* and schizophrenia. We performed a case–control analysis to investigate the association between *EP400* deleterious mutations and schizophrenia. The single-variant association test indicated that deleterious variants in *EP400* were not significantly associated with schizophrenia. Moreover, in gene-based rare-variant association tests, *EP400* did not show significant enrichment of rare variants in schizophrenia. These results, together with the absence of the identified familial mutation (rs200626129) in 285 schizophrenia patients, suggest that rare variants in *EP400* do not account for a large subgroup of schizophrenia patients.

Recent investigations have demonstrated the extreme difficulty of identifying individual genes with significant associations in rare-variant association studies^17,18^. For example, Genovese et al.^55^ failed to implicate any specific gene (including *EP400*) in schizophrenia, and only one exome study to date, which investigated approximately 15,000 exomes, has been successful in reporting an individual risk gene (*SETD1A*) for schizophrenia^15^. Given these findings, the present study likely lacked the statistical power needed to support rare-variant accumulation because of its small sample size. Our study concept was based on the rare variants’ contribution with relatively high impact effect; thus, a large sample size is necessary for rare-variant association studies^56^. To explore the association between rare *EP400* variants and schizophrenia via rare-variant association studies, further analyses with larger sample sizes (>10,000, with multi-ethnic populations) are required.

Ep400-p.P2715L mice, corresponding to the human P2805L mutation, showed anxiety-like behaviour and had decreased axon diameters in the spinal cord white matter. These results reflect the successful use of mice to demonstrate that the mouse P2715L mutation causes histological and neurobehavioural abnormalities, which may help to explain the pathogenesis of the human P2805L mutation in the family that was investigated in the present study. Anxiety is not a main symptom of schizophrenia but is sometimes observed in patients with this disease^57,58^. In addition, some studies have reported myelin and axon abnormalities in the white matter of schizophrenia patients^59,60^. These reports, therefore, indicate that our mouse model may mimic at least some of the biological aetiologies of this family. However, the phenotypes observed in *Ep400* gene-edited mice in the present study are not likely to be easily generalisable, especially considering that the burden of rare variants in *EP400* on the development of schizophrenia remains unclear. In addition, because the present study was limited to screening only, it is necessary to analyse whether there are any unknown phenotypes in these mice, and whether they show appropriate phenotypes to be considered a model of schizophrenia.

EP400 is the central ATP-hydrolysing subunit of the TIP60/EP400 complex^61,62^, which is thought to exchange histone H2A for H2A.Z in nucleosomes (especially in gene regulatory regions such as promoters^63–65^) to influence gene expression and DNA repair^66^. However, the molecular functions of EP400 in the CNS are poorly characterised. Elsesser et al. reported that EP400 interacts with the transcription factor SOX10, binds to myelin regulatory factor (MYRF) gene regulatory regions, and induces this central transcriptional regulator of myelination^52^. Mutual interactions between oligodendrocytes and neurons control the myelination and maturation of neural axons^67–69^. Accordingly, the p.P2715L mutation might decrease axon diameter via the dysfunction of these mutual interactions between oligodendrocytes and neurons in the developing CNS. In addition, the p.P2715L mice partially replicated the phenotype of *Ep400* conditional-knockout mice that had been reported previously. This result, together with the finding that p.P2715L mice show a novel phenotype—of reduced axon diameter—suggests that the p.P2715L mutation may not simply cause a loss of function of the EP400 protein (e.g., a gain-of-function or partial-loss-of-function mutation). Further functional studies that confirm the abnormal functions of mutated EP400 in the developing CNS are, therefore, required to reveal the underlying pathogenic mechanisms.

In conclusion, we identified a unique missense variant in *EP400* through the exome sequencing of a Japanese family containing multiple schizophrenia patients. The histological and neurobehavioural abnormalities observed in *Ep400* gene-edited mice implicate the pathogenesis of this familial mutation in the schizophrenia multiplex family. The phenotypes of the *Ep400* gene-edited mice may not be generalisable, because the burden of rare variants in *EP400* on the development of schizophrenia remains unclear. However, a better understanding of the functional effects of EP400 in the CNS might help to clarify both the pathophysiology of schizophrenia and the development of new therapeutic targets for this disease.

## Supplementary information

S_Video 1

S_Video 2

Supplementary Information

## Data Availability

All relevant data supporting the findings of this study are available within the article and its Supplementary Information files, or from the corresponding authors upon reasonable request. Source data are provided with this paper.

## Source data

Source data
