# Supplementary material for: A unique missense variant in the E1A-binding protein P400 gene is implicated in schizophrenia by whole-exome sequencing and mutant mouse models
Source: Transl Psychiatry. 2021 Feb 18;11:132. doi: 10.1038/s41398-021-01258-1 (PMC7892873; doi:10.1038/s41398-021-01258-1)
Supplement: Supplementary file 3 — Supplementary Information [file 41398_2021_1258_MOESM3_ESM.docx]

Supplementary Information for

**A unique missense variant in** **the E1A-binding protein P400 gene is implicated in schizophrenia by whole-exome sequencing and mutant mouse models**

Morimoto et al.

**Supplementary information**

**Supplementary Figure 1:** Immunofluorescent staining of CNS tissue from Ep400-p.P2715L mice

**Supplementary Figure 2:** Phenotypes of Ep400-p.P2715L#2 mice

**Supplementary Figure 3:** HE staining of CNS tissue from Ep400-p.P2715L mice

**Supplementary Table 1:** Mean depth and coverage rate of WES

**Supplementary Table 2:** Segregation of SNVs with schizophrenia

**Supplementary Table 3:** WES variant filtering

**Supplementary Table 4:** All rare variants and allele counts in case–control samples

**Supplementary Table 5:** Mendelian ratios from interbreeding of EP400-p.P2715L#1/+ mice

**Supplementary Table 6:** Body weights of Ep400-p.P2715L#1 mice

**Supplementary Table 7:** Hindlimb clasping of Ep400-p.P2715L#1, 2 mice

**Supplementary Video 1:** Hindlimb clasping sign

**Supplementary Video 2:** Stretched approaches to stranger mice and active exploration in the social interaction test


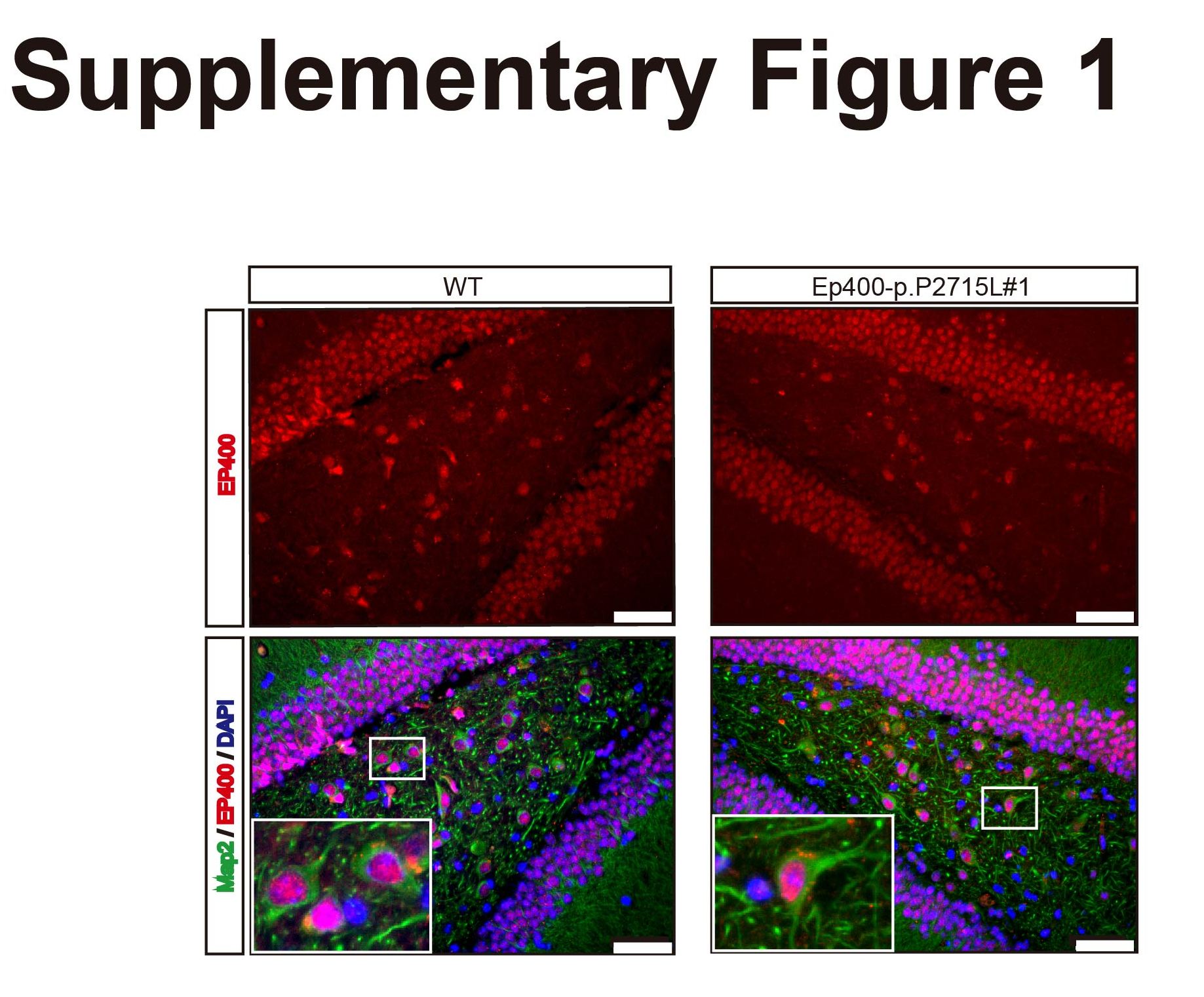


**Supplementary Figure 1: Immunofluorescent staining of CNS tissue from Ep400-p.P2715L mice.**

Immunofluorescent staining of the hippocampal dentate gyrus. White box: EP400 expression in neural cells. Red: EP400. Green: MAP2, a neuronal marker. Blue: DAPI. White scale bar = 50 µm.

**
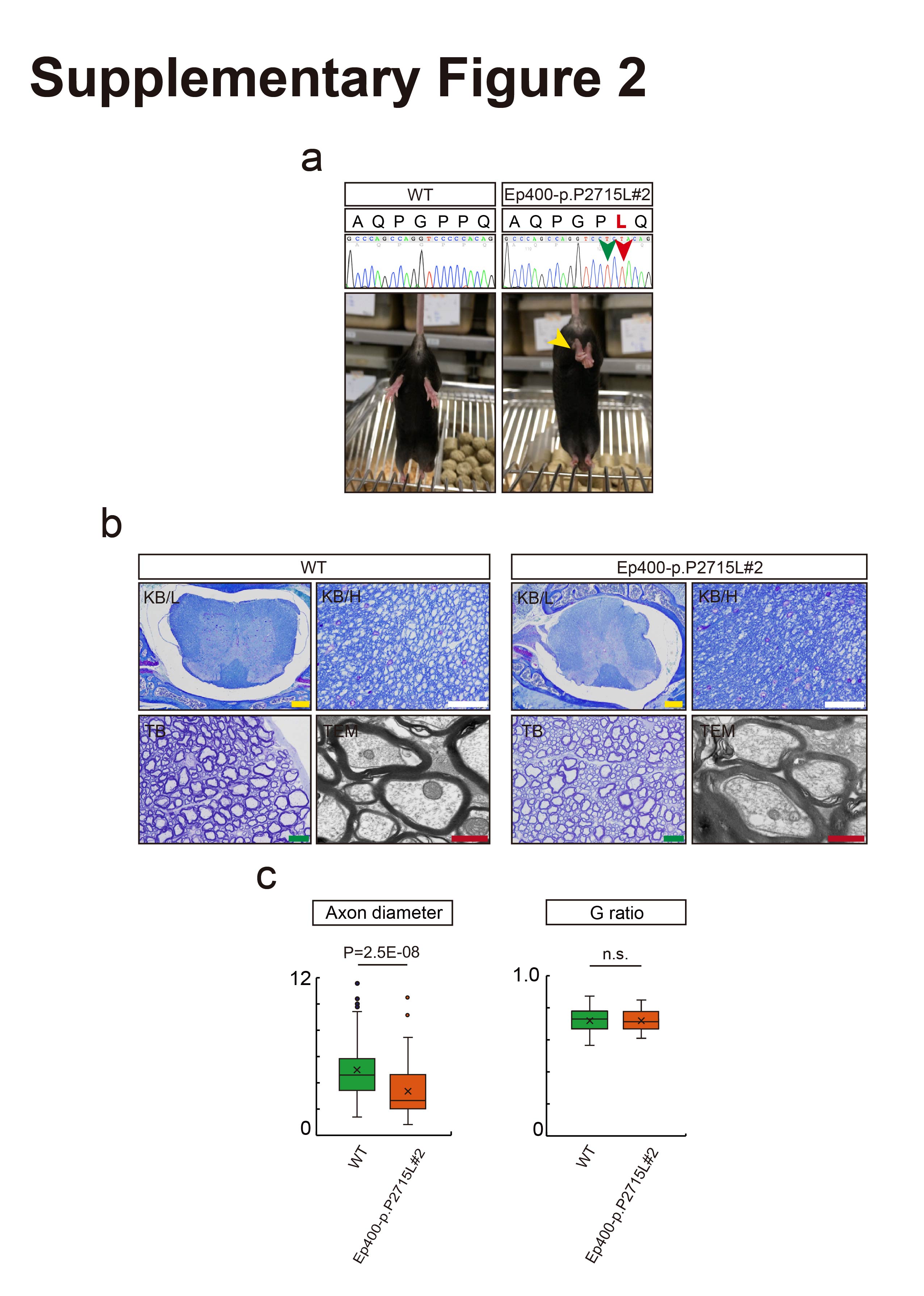
**

**Supplementary Figure 2: Phenotypes of Ep400-p.P2715L#2 mice.**

a. Genotype and hindlimb clasping. Sanger sequencing was used to confirm the missense mutation (p.P2715L, red arrow) and synonymous mutation to break the protospacer adjacent motif (PAM; green arrow) in *Ep400*. Hindlimb clasping (yellow arrow) was used as a marker of CNS dysfunction (p.P2715L#2 mice: *n* = 4; WT: *n* = 6). b. KB and TB staining was used to investigate axon diameter in the spinal marrow in 3-month-old WT and Ep400-p.P2715L#2 mice. TEM analysis was used to investigate morphological abnormalities in myelin sheaths (p.P2715L#2 mice: *n* = 2; WT: *n* = 2). KB/L= Klüver–Barrera staining, in a low-power field. KB/H= Klüver–Barrera staining, in a high-power field. TB = Toluidine blue staining. Yellow scale bar = 200 µm. White scale bar = 50 µm. Green scale bar = 10 µm. Red scale bar = 1 µm. c. TEM analysis was used to compare axon diameters and G-ratios between Ep400-p.P2715L#2 and WT mice. A significant decrease in axon diameter in Ep400-p.P2715L#2 mice was confirmed by TEM analysis. Ep400-p.P2715L#2 mice did not show significant differences in G-ratios compared with WT littermates (p.P2715L#2 mice: *n* = 2; WT: *n* = 2).


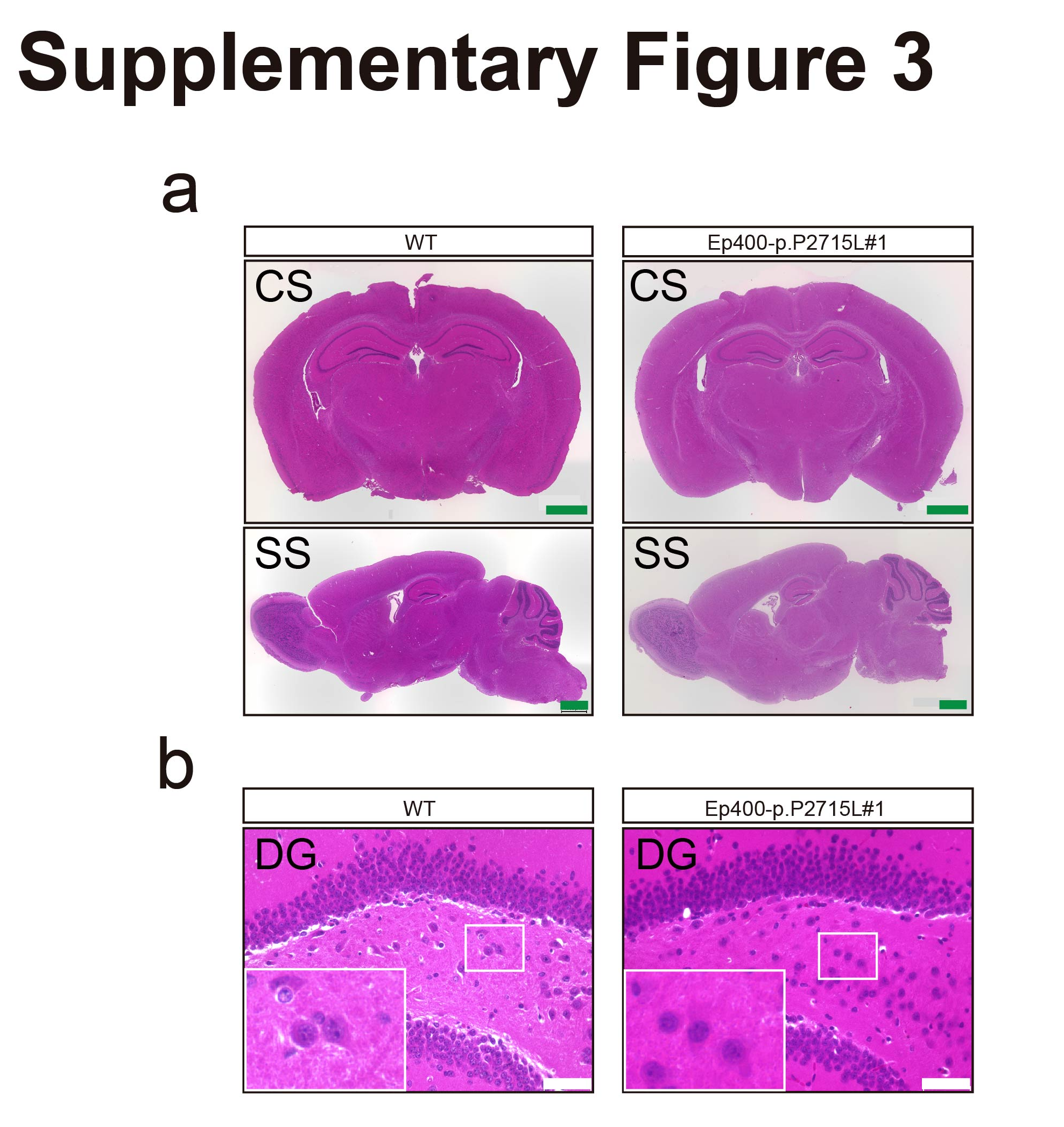


**Supplementary Figure 3: HE staining of CNS tissue from Ep400-p.P2715L mice.**

a. HE staining was used to investigate morphological changes in the brains of Ep400-p.P2715L#1 and WT mice (p.P2715L#1 mice: *n* = 6; WT: *n* = 6). CS = coronal section, SS = sagittal section. Green scale bar = 1 000 µm. b. HE staining in the hippocampal dentate gyrus. DG = hippocampal dentate gyrus. White box: morphologies of the neuronal cell bodies. Green scale bar = 1 000 µm.

| **Supplementary Table 1. Mean depth and coverage rate of WES** | | | | |  |  |  |
| --- | --- | --- | --- | --- | --- | --- | --- |
| **Sample** | **Total_bp** | **Mean** | **Median** | **> 5×** | **> 10×** | **> 20×** | **> 40×** |
| I-9 | 5 780 931 630 | 114.84 | 103 | 98.4 | 97.8 | 95.9 | 87.7 |
| I-13 | 4 485 437 332 | 89.1 | 79 | 98.3 | 97.4 | 94.2 | 81.4 |
| II-3 | 3 717 584 527 | 73.85 | 66 | 98.1 | 96.8 | 92.3 | 74.9 |
| II-5 | 4 849 139 484 | 96.33 | 87 | 98.3 | 97.5 | 94.7 | 83.5 |
| II-11 | 5 485 544 547 | 108.97 | 97 | 98.5 | 97.8 | 95.7 | 86.9 |
| II-12 | 4 939 691 022 | 98.13 | 89 | 98.2 | 97.5 | 94.8 | 83.9 |
| II-17 | 5 519 231 028 | 109.64 | 99 | 98.3 | 97.7 | 95.6 | 86.8 |
| II-20 | 4 116 091 715 | 81.77 | 73 | 98.3 | 97.2 | 93.4 | 78.6 |
| III-2 | 2 873 164 713 | 57.08 | 51 | 97.8 | 95.8 | 87.7 | 62.7 |
| III-3 | 5 105 509 447 | 101.42 | 91 | 98.2 | 97.5 | 95.0 | 84.7 |

|  |  |  |  |  |  |
| --- | --- | --- | --- | --- | --- |

| **Supplementary Table 2: Segregation of SNVs with schizophrenia** | | | | | | | |  | |  | |  |
| --- | --- | --- | --- | --- | --- | --- | --- | --- | --- | --- | --- | --- |
| **Gene** | **Variant** | | **Allele frequency in public databases** | | | | | | **Mutation allele counts in case–control samples** | | | |
|  |  |  | **HGVD** | | **4.7KJPN** | | **gnomADexome** | | **Schizophrenia (*n* = 570)** | | **Control (*n* = 890)** | |
| *SBNO1* | chr12:123311084;T>C | | 0.00124 | | 0.0012 | | 7.958e-06 | | 0 | | 2 | |
| *EP400* | chr12:132064747;C>T | | none | | none | | 2.415e-05 | | 0 | | 0 | |
|  |  | |  | |  | |  | |  | |  | |
|  | |  | |  | |  | |  | |  | |  |

| **Supplementary Table 3: WES variant filtering** | |
| --- | --- |
| **WES variant filtering** | **Number of variants** |
| 1, Total variants in 10 family members | 148 069 |
| 2, Stop-gain, stop-loss, nonsynonymous, or splice-site mutations | 18 825 |
| 3, MAF ≤ 0.5% in HGVD2.30, 4.7KJPN, gnomADexome | 2 176 |
| 4, Unique heterozygous variants | 164 |
| 5, Specific to SCZ/TRS family members | 2 |
| 6, Novel variants in 445 healthy Japanese controls | 1 |
| WES: whole-exome sequencing; MAF: minor allele frequency; SCZ: schizophrenia; TRS: treatment-resistant schizophrenia. | |

| **Supplementary Table 4: All rare variants and allele counts in case–control samples** | | | | | |  |  | |  |  | |
| --- | --- | --- | --- | --- | --- | --- | --- | --- | --- | --- | --- |
| **Variant** | **cDNA change** | **Protein change** | **4.7KJPN** | **HGVD2.30** | **gnomADexome** | **SCZ**  **(*n* = 570)** | | **Control**  **(*n* = 890)** | ***P*-value** | |  |
| chr12:131960919-131960920;CT>- | c.300_301del | p.T100fs | none | none | none | 0 | 1 | | 1 | |  |
| chr12:131990048;C>G | c.2494C>G | p.R832G | 0.0017 | none | none | 1 | 0 | | 0.3904 | |  |
| chr12:132005068;C>T | c.2827-9C>T | none | 0.0001 | none | none | 1 | 0 | | 0.3904 | |  |
| chr12:132006129;G>C | c.2953G>C | p.G985R | 0.0034 | 0.0045 | 3.183e-05 | 1 | 1 | | 1 | |  |
| chr12:132006216;G>A | c.3040G>A | p.G1014R | 0.0005 | 0.0012 | 3.181e-05 | 1 | 0 | | 0.3904 | |  |
| chr12:132013032;C>A | c.3465C>A | p.F1155L | none | none | none | 0 | 1 | | 1 | |  |
| chr12:132017572;G>A | c.3961G>A | p.V1321I | none | none | none | 1 | 0 | | 0.3904 | |  |
| chr12:132018316;A>C | c.4217A>C | p.E1406A | none | none | none | 1 | 0 | | 0.3904 | |  |
| chr12:132020123;G>A | c.4352G>A | p.R1451Q | 0.0005 | 0.0004 | 8.505e-05 | 0 | 1 | | 1 | |  |
| chr12:132020126;C>T | c.4355C>T | p.T1452M | 0.0032 | 0.0029 | 0.0002 | 4 | 0 | | 0.0231 | |  |
| chr12:132021127;G>A | c.4496G>A | p.R1499Q | 0.0003 | none | 2.118e-05 | 1 | 0 | | 0.3904 | |  |
| chr12:132028282;T>C | c.5375T>C | p.I1792T | 0.0006 | 0.0012 | 0.0004 | 1 | 4 | | 0.6541 | |  |
| chr12:132029721;C>T | c.5402C>T | p.P1801L | none | none | 2.009e-05 | 0 | 1 | | 1 | |  |
| chr12:132044234;G>A | c.6508G>A | p.E2170K | 0.0026 | 0.0009 | 5.571e-05 | 2 | 0 | | 0.1523 | |  |
| chr12:132045766;A>G | c.7066A>G | p.I2356V | none | none | 9.149e-05 | 1 | 0 | | 0.3904 | |  |
| chr12:132053494;T>C | c.7625T>C | p.V2542A | 0.0006 | 0.0013 | 1.443e-05 | 1 | 1 | | 1 | |  |
| chr12:132062186;C>T | c.7961C>T | p.A2654V | none | none | 0.0007 | 0 | 2 | | 0.5237 | |  |
| chr12:132062596;G>T | c.8229G>T | p.Q2743H | 0.0006 | 0.0004 | 6.995e-05 | 2 | 0 | | 0.1523 | |  |
| chr12:132064789;C>T | c.8456C>T | p.P2819L | none | none | 7.713e-05 | 1 | 0 | | 0.3904 | |  |
| chr12:132064836;G>A | c.8503G>A | p.G2835S | 0.0002 | 0.0004 | 2.068e-05 | 0 | 1 | | 1 | |  |
| chr12:132066851;G>T | c.8631G>T | p.L2877F | none | none | none | 1 | 0 | | 0.3904 | |  |
| chr12:132067475;G>A | c.8863G>A | p.V2955I | none | none | 4.065e-05 | 0 | 1 | | 1 | |  |
| chr12:132069582;A>G | c.8962A>G | p.T2988A | none | none | none | 1 | 0 | | 0.3904 | |  |
| *P*-values were calculated using Fisher’s exact test. The significance threshold was 0.002174, with Bonferroni correction. SCZ:schizophrenia. | | | | | | | | | | |  |

| **Supplementary Table 5: Mendelian ratios from interbreeding of EP400-p.P2715L#1/+ mice** | | | | |
| --- | --- | --- | --- | --- |
| **Genotype** | **Observed number** | **Expected number** | **Chi-squared value** | ***P*-value** |
| Ep400-WT/WT | 18 | 19 | - | - |
| Ep400-p.P2715L#1/WT | 41 | 38 | 0.2119 | 0.5906 |
| Ep400-P2715L#1/P2715L#1 (Ep400-p.P2715L#1) | 17 | 19 | 0.0286 | 0.608 |
| Total | 76 | 76 | 0.5 | 0.7788 |

| **Supplementary Table 6: Body weights of Ep400-p.P2715L#1 mice** | | |
| --- | --- | --- |
| **Animal ID** | **Body weight (g) at 3 months** | **Body weight (g) at 6 months** |
| WT-01 | 24.4 | 29.7 |
| WT-02 | 27.3 | 35.7 |
| WT-03 | 27.0 | 32.2 |
| WT-04 | 27.2 | 36.3 |
| WT-05 | 26.9 | 30.5 |
| WT-06 | 29.8 | 40.3 |
| Ep400-p.P2715L#1-01 | 28.4 | 37.4 |
| Ep400-p.P2715L#1-02 | 26.9 | 36.8 |
| Ep400-p.P2715L#1-03 | 31.7 | 41.5 |
| Ep400-p.P2715L#1-04 | 30.3 | 40.1 |
| Ep400-p.P2715L#1-05 | 28.3 | 34.2 |
| Ep400-p.P2715L#1-06 | 27.2 | 38.3 |

| **Supplementary Table 7: Hindlimb clasping of Ep400-p.P2715L#1, 2 mice** | |
| --- | --- |
| **Animal ID** | **Hindlimb clasping (3 months)** |
| WT-H-01 | 0 |
| WT-H-02 | 1 |
| WT-H-03 | 2 |
| WT-H-04 | 0 |
| WT-H-05 | 0 |
| WT-H-06 | 0 |
| Ep400-p.P2715L#1-H-01 | 3 |
| Ep400-p.P2715L#1-H-02 | 2 |
| Ep400-p.P2715L#1-H-03 | 1 |
| Ep400-p.P2715L#1-H-04 | 3 |
| Ep400-p.P2715L#1-H-05 | 2 |
| Ep400-p.P2715L#1-H-06 | 0 |
| Ep400-p.P2715L#2-H-01 | 2 |
| Ep400-p.P2715L#2-H-02 | 2 |
| Ep400-p.P2715L#2-H-03 | 3 |
| Ep400-p.P2715L#2-H-04 | 1 |

**Supplementary Video 1:** Hindlimb clasping sign. Abnormal hindlimb clasping was used as a marker of CNS dysfunction.

**Supplementary Video 2:** Stretched approaches to stranger mice and active exploration in the social interaction test.
